# Smoking and timing of cessation on postoperative pulmonary complications after curative-intent lung cancer surgery

**DOI:** 10.1186/s13019-017-0614-4

**Published:** 2017-06-19

**Authors:** Sebastian T. Lugg, Theofano Tikka, Paula J. Agostini, Amy Kerr, Kerry Adams, Maninder S. Kalkat, Richard S. Steyn, Pala B. Rajesh, Ehab Bishay, David R. Thickett, Babu Naidu

**Affiliations:** 1 Centre for Translational Inflammation Research, Institute of Inflammation and Ageing, University of Birmingham, Birmingham, B15 2WB UK; 2 Department of Thoracic Surgery, Heart of England NHS Foundation Trust, Bordesley Green East, Birmingham, UK

**Keywords:** Thoracic surgery, Pneumonia, Atelectasis, Smoking

## Abstract

**Background:**

Smoking is a risk factor for postoperative pulmonary complications (PPC) following non-small cell lung cancer (NSCLC) surgery. The optimal timing for preoperative smoking cessation has not been identified. Our study aimed to observe the impact of preoperative smoking cessation on PPC incidence and other postoperative outcomes including long-term survival.

**Methods:**

A prospective study included consecutive patients following resection for NSCLC in a regional thoracic centre over a 4-year period (2010–2014). Patients were stratified according to self-reported preoperative smoking status. The primary endpoint was PPC incidence, which was assessed from postoperative day one onwards using the Melbourne Group Scale. Secondary endpoints included short-term outcomes (hospital length of stay [LOS], intensive therapy unit [ITU] admission, 30-day hospital readmission rate) and long-term survival.

**Results:**

Four hundred and sixty-two patients included 111 (24%) current smokers, 55 (12%) ex-smokers <6 weeks, 245 (53%) ex-smokers ≥6 weeks and 51 (11%) never smokers. PPC occurred in 60 (13%) patients in total. Compared to never smokers, current smokers had a higher frequency of PPC (22% vs. 2%, *p* = 0.004), higher frequency of ITU admission (14% vs. 0%; *p* = 0.001) and a longer median (IQR) hospital LOS (6 [5] vs. 5 [2]; *p* = 0.001). In the ex-smokers there was a trend for a lower frequency of PPC (<6 weeks, 10.9% vs. ≥6 weeks, 11.8%) and ITU admission (<6 weeks, 5.5% vs. ≥6 weeks, 4.5%), but there was no difference between the <6 weeks or ≥6 weeks ex-smoking groups prior to surgery. There was no significant difference in long-term survival found between the groups of differing smoking status (median follow-up 29.8 months, 95%CI 28.4–31.1).

**Conclusion:**

Current smokers have higher postoperative morbidity; this risk reduces following smoking cessation but 6 weeks does not appear to identify a time-point where differences in outcomes are noted.

## Background

Non-small-cell lung cancer (NSCLC) represents approximately 85% of all lung cancer cases. In early-stages, surgery remains the corner stone of treatment, but is complicated by a high risk of developing a postoperative pulmonary complication (PPC), of which atelectasis and pneumonia are most common. Developing a PPC has been shown to increase in short-term complications including increased hospital mortality (0.5% to 12%), ITU admission rate (1.5% to 26%) and hospital length of stay (LOS) (5 to 14 days) [1]. Patients who develop a PPC also have a poorer long-term outcome with a 6-month reduction in mean overall survival [2]. We have previously demonstrated that smoking is the strongest independent risk factor for developing a PPC after thoracic surgery [1–2]. Furthermore, smoking has been shown to have a negative impact on long-term overall survival and disease progression in lung cancer patients following curative-intent surgery [3].

Risk modification is via preoperative smoking cessation. Few studies have suggested that smoking abstinence of at least 4 weeks may be necessary for patients undergoing thoracic surgery in order to reduce the incidence of PPC and hospital mortality [4, 5]. Despite this, an optimal duration of preoperative smoking cessation has not been identified. Many studies addressing this question are retrospective in nature with small sample sizes, the definition and criteria of PPC varies between studies and there is a paucity of published studies demonstrating the effects of preoperative smoking cessation on long-term survival.

To address these issues, the primary aim of this study was to prospectively observe the timing of preoperative smoking cessation on PPC incidence following curative-intent lung cancer surgery. The secondary aim of this study was to follow up these patients to ascertain if an optimal interval of preoperative smoking cessation is identifiable to improve the short-term postoperative outcome and long-term survival.

## Methods

### Patient selection

This prospective observational study included all patients who underwent curative-intent lung resection for NSCLC via open thoracotomy or video-assisted thoracoscopic surgery (VATS) in a large UK thoracic centre between April 2010 and April 2014. The study received ethics approval by the National Research Ethics Service Committee West Midlands, Edgbaston. Decisions regarding patient selection and operability were informed by the British Thoracic Society guidelines [6]. Preoperative smoking status was categorised into current smokers who continued up to the date of surgery; ex-smokers who stopped smoking <6 weeks prior to surgery, ex-smokers who stopped smoking ≥6 weeks prior to surgery, and never smokers. Six weeks was chosen as the reporting period as previous evidence suggested that preoperative smoking cessation interventions might be more beneficial when offered within this timeframe, rather than immediately before surgery [7]. Smoking data was collected by patients self-reporting to the specialist thoracic research team (including nurses and physicians) at the preoperative assessment and the hospital admission using a paper based case report form, which was then uploaded onto the electronic database.

All operations were performed with single lung ventilation under general anaesthesia. Patients were subsequently scheduled for extubation in the operating room and postoperatively managed in a thoracic high-dependency unit (HDU) (level 2) and specialist ward unless complications required their admission to the ITU. The choice of postoperative analgesia was agreed between the anaesthetist and patient. From postoperative day one, all patients received daily physiotherapy comprising of deep breathing exercises, incentive spirometry, supported coughing and mobilisation.

All patients included in the study had a confirmed pathologically staged diagnosis of NSCLC (TNM 7^th^ edition). Baseline data included demographics, body mass index (BMI), percentage predicted forced expiratory volume in one second (FEV_1_), American Society of Anaethesiologists (ASA) score and subjective preoperative physical activity level. Comorbidities including cardiovascular disease COPD diagnosis were both defined by the referring physician, with COPD severity staged according to percentage predicted FEV_1_.

### Primary endpoint

The Melbourne Group Scale (MGS) is a standardised scoring system previously validated by our group to define the presence/absence of a PPC [1]. The MGS defines PPC when patients present with four or more of the following eight factors: chest x-ray (CXR) findings of atelectasis or consolidation, raised white blood cell count (WBC) (>11.2^9^/l) or administration of antibiotics (in addition to prophylactic antibiotics), temperature >38 °C; signs of infection on sputum microbiology, purulent sputum differing from preoperative status, oxygen saturations <90% on room air, physician diagnosis of chest infection/pneumonia, and prolonged HDU stay or readmission to HDU or ITU for respiratory complications. The MGS was used daily from postoperative day one onwards by senior physiotherapists who were performing their routine respiratory assessments. CXR results and physician diagnoses of chest infection/pneumonia were confirmed by a senior thoracic surgeon.

### Secondary endpoints

Postoperative data included hospital LOS, ITU LOS, ITU admission frequency and 30-day hospital readmission rates (secondary to surgical or pulmonary complications). All patients were followed up during the study period for overall survival; defined as the time from date of surgery to the date of death or censored at the last date of confirmed living status. Cause of death was obtained from the death certificate and hospital records. Deaths were classified as a postoperative complication for those who died within the initial hospital admission or within 30-days of surgery, cancer related for patients who died of disease progression or recurrence, non-cancer related from patients who died of other causes, or cause of death unknown where records were not available.

### Statistical analysis

Results are expressed as mean (95%CI) or median (IQR) for continuous variables depending on normality of data and as a percentage for categorical variables. Univariate analysis of risk factors using Pearson’s chi-square test or Fisher’s exact test where appropriate for categorical variables, and Kruskal-Wallis test or ANOVA for continuous variables were performed following the assessment of normality of data.

Significant predictors of survival were identified using Proportional Cox regression modeling. Covariates were assessed for validity of proportional hazard assumption prior to inclusion in the model. Standardized score process and cumulative martingale residuals plots were generated. The following covariates were well specified and met the proportional hazard assumption: stage, age, PPC, total LOS, ITU LOS, VATS surgery, hypertension, diabetes, ischaemic heart disease, COPD, FEV_1_ % predicted, sex, type of analgesia, preoperative physical activity level and smoking status. The statistical significant covariates were identified following a backward elimination process. The level of significance was set at 0.05. Predictive survival curves adjusted for the different smoking status categories were generated based on the final Cox proportional hazards model at different levels of the predictor variables. All analyses were performed using the SAS statistical package version 9.3 (SAS Institute, Inc, Carry NC) and IBM SPSS version 20.0.

## Results

### Study population

There were 462 patients with NSCLC who underwent pulmonary resection during the study period of which 277 (60%) were male. The mean ± SD age of the group was 68.8 ± 8.5 years. The mean predicted FEV_1_ was 79.2 ± 20.4%, mean BMI was 26.8 ± 8.9 kg/m^2^, 237 (51.3%) had an ASA score ≥3 and 136 (29.4%) had COPD. There were 111 (24%) current smokers, 55 (12%) ex-smokers <6 weeks, 245 (53%) ex-smokers ≥6 weeks, and 51 (11%) never smokers.

The most frequent procedures were lobectomy (*n* = 361, 78.1%), followed by wedge or segmentectomy (*n* = 61, 13.2%), pneumonectomy (*n* = 32, 6.9%), sleeve resections (*n* = 6, 1.3%) and chest wall resection/reconstruction (*n* = 2, 0.4%). VATS approach was used in 39 patients (8.4%). Patients were NSCLC staging of I (*n* = 229, 50%), II (*n* = 147, 32%), IIIA (76, 16%) and IIIB (*n* = 6, 1%).

Age, FEV_1_% predicted and BMI were all significantly lower (*p* < 0.05) in current smokers than never smokers. There were significantly more males and patients with COPD diagnosis in the current smoking group (*p* < 0.05, Table 1); though there were no significant differences in the severity of COPD between groups. Cancer staging, preoperative physical activity level, ASA score and diagnosis of cardiovascular disease were not significantly associated with smoking.

**Table 1:** Results of univariate analysis to determine factors associated with smoking status

		Current smokers	Ex-smokers <6 weeks	Ex-smokers ≥6 weeks	Never smokers	*p*-value
		(*n* = 111)	(*n* = 55)	(*n* = 245)	(*n* = 51)	
Mean (95%CI) age (years)		67 (65–68.3)^f^	68 (65.6–9.9)^e,f^	70 (68.8–70.8)^e^	72 (67.2–72.8)^e,f^	0.006^a^
Median (IQR) BMI (kg/m^2^)		24.1 (6.3)^f^	24 (7.5)^e^	26.9 (5.4)^e,f^	26 (6.4)^e,f^	<0.001^b^
Mean (95%CI) FEV_1_% predicted		72 (68.6–75.7)^g^	78 (73.5–81.8)^f,g^	79 (76.6–81.8)^e,f^	94 (87.4–100.5)^e^	<0.001^a^
COPD		50.4% (*n* = 56)^g^	20.0% (*n* = 11)^f,g^	26.9% (*n* = 66)^e,f^	5.9% (*n* = 3)^e^	<0.001^c^
COPD Severity (FEV_1_% predicted)	Mild (≥80)	18.9% (*n* = 21)	3.6% (*n* = 2)	6.5% (*n* = 16)	2% (*n* = 1)	0.358^d^
	Moderate (≥50 to <80)	23.4% (*n* = 26)	16.4% (*n* = 9)	15.5% (*n* = 38)	3.9% (*n* = 2)	
	Severe (≥30 to <50)	8.1% (*n* = 9)	0%	4.5% (*n* = 11)	0%	
Male sex		63.9% (*n* = 71)^f^	60.0% (*n* = 33)^e,f^	63.2% (*n* = 155)^f^	35.2% (*n* = 18)^e^	0.002^c^
Ischaemic heart disease		14.4% (*n* = 16)	18.1% (*n* = 10)	15.1% (*n* = 37)	7.8% (*n* = 4)	0.476^c^
Diabetes		12.6% (*n* = 14)	5.5% (*n* = 3)	13.1% (*n* = 32)	11.7% (*n* = 6)	0.503^d^
Hypertension		42.3% (*n* = 47)	41.8% (*n* = 23)	45.3% (*n* = 111)	52.9% (*n* = 27)	0.604^c^
ASA score ≥3		58.5% (*n* = 65)	52.7% (*n* = 29)	49.8% (*n* = 122)	41.2% (*n* = 21)	0.195^c^
Preoperative activity level <400 m		29.4% (*n* = 32/109)	25% (*n* = 13/52)	29.7% (*n* = 71/239)	21.6% (*n* = 11/51)	0.634^c^
Staging	I	54.1% (*n* = 60)	47.3% (*n* = 26)	48.2% (*n* = 118)	49.0% (*n* = 25)	0.36 ^d^
	II	29.7% (*n* = 33)	32.7% (*n* = 18)	34.7% (*n* = 85)	21.6% (*n* = 11)	
	IIIA	14.4% (*n* = 16)	18.2% (*n* = 10)	15.9% (*n* = 39	21.6% (*n* = 11)	
	IIIB	0.90% (*n* = 1)	0%	0.41% (*n* = 1)	3.9% (*n* = 2)	
VATS	No	91.9% (*n* = 102)	85.5% (*n* = 47)	92.7% (*n* = 227)	92.2% (*n* = 47)	0.396^d^
	Yes	8.1% (*n* = 9)	14.5% (*n* = 8)	7.3% (*n* = 18)	7.8% (*n* = 4)	
Surgery	Lobectomy	81.1% (*n* = 90)	78.2% (*n* = 43)	77.6% (*n* = 190)	74.5% (*n* = 38)	0.592^d^
	Subsegmectomy/wedge	10.8% (*n* = 12)	9.1% (*n* = 5)	14.3% (*n* = 35)	17.6% (*n* = 9)	
	Pneumonectomy	5.4% (*n* = 6)	10.9% (*n* = 6)	6.5% (*n* = 16)	7.8% (*n* = 4)	
	Sleeve	1.8% (*n* = 2)	0%	1.6% (*n* = 4)	0%	
	Chest wall	0.9% (*n* = 1)	1.8% (*n* = 1)	0%	0%	
Analgesia	Epidural	40.5% (*n* = 45)	29.0% (*n* = 16)	37.1% (*n* = 91)	43.1% (*n* = 22)	0.217^d^
	Intrathecal morphine	23.4% (*n* = 26)	36.4% (*n* = 20)	21.6% (*n* = 53)	21.6% (*n* = 11)	
	Morphine infusion	11.7% (*n* = 13)	10.9% (*n* = 6)	8.6% (*n* = 21)	5.9% (*n* = 3)	
	PCA	5.4% (n = 6)	7.3% (*n* = 4)	5.3% (*n* = 13)	5.9% (*n* = 3)	
	Paravertebral	18.9% (*n* = 21)	12.7% (*n* = 7)	26.5% (*n* = 65)	23.5% (*n* = 12)	
	Other	0%	3.6% (*n* = 2)	0.8% (*n* = 2)	0%	

*BMI* body mass index, *FEV*_*1*_ forced expiratory volume in one second, *COPD* chronic obstructive pulmonary disease, *ASA* American Society of Anaethesiologists, *VATS* Video-assisted thoracoscopic surgery, *PCA* patient controlled analgesia.^a^ ANOVA with Bonferroni post-hoc analysis;^b^ Kruskal-Wallis test with Dunn Bonferroni post-hoc multiple comparison test;^c^ Chi-sqaure test with Bonferroni post-hoc analysis;^d^ Fisher’s Exact test with Bonferroni post-hoc analysis. When overall *p*-value is less than 0.05; each subscript letter(^e-g^) denotes a subset of smoking category whose column proportion does not differ significantly from each other (Bonferroni post-hoc multiple comparison test)

### PPC incidence

PPC was present in 60 patients (13%); the most common positive factors to trigger a score of 4 were raised WBC (*n* = 54, 11.7%), purulent sputum (*n* = 48, 10.4%), CXR findings (*n* = 48, 10.4%), and reduced oxygen saturations (*n* = 48, 10.4%). Compared to never smokers, current smokers had a higher frequency of PPC (22% vs. 2%, *p* = 0.004) (Table 2). Compared to current smokers, in the ex-smoking groups there was a trend for a lower frequency of PPC (<6 weeks, 10.9% vs. ≥6 weeks, 11.8%) but there was no significant differences between ex-smoking groups, or when compared to current and never smokers.

**Table 2:** Morbidity and mortality in patients stratified by smoking status

		Current smokers	Ex-smokers <6 weeks	Ex-smokers ≥6 weeks	Never smokers	*p*-value
		(*n* = 111)	(*n* = 55)	(*n* = 245)	(*n* = 51)	
PPC Frequency		21.6% (*n* = 24)^d^	10.9% (*n* = 6)^c,d^	11.8% (*n* = 29)^c,d^	2.0% (*n* = 1)^c^	0.004^a^
ITU admission		14.4% (*n* = 16)^d^	5.5% (*n* = 3)^c,d^	4.5% (*n* = 11)^c,d^	0%^c^	0.001^b^
Median (IQR) LOS (days)		6 (5)^d^	5 (3)^c,d^	6 (4)^c,d^	5 (2)^c^	0.001^f^
Median (IQR) ITU LOS (days)		4 (10) (*n* = 16)^e^	2 ( ^*^ ) (*n* = 3)^c,d,e^	3 (2) (*n* = 11)^c,d^	0 (0) (*n* = 0)^c^	0.002^f^
30-day hospital readmissions		13.6% (*n* = 15)	14.8% (*n* = 8)	15.1% (*n* = 36)	7.8% (*n* = 4)	0.793^a^
Mortality	Total	30.6% (*n* = 34)	32.7% (*n* = 18)	30.2% (*n* = 74)	23.5% (*n* = 12)	0.744^a^
	30-day	1.8% (*n* = 2)	0%	2.4% (*n* = 6)	2.0% (*n* = 1)	0.874^b^
	90-day	5.4% (*n* = 6)	1.8% (*n* = 1)	3.2% (*n* = 8)	5.9% (*n* = 3)	0.575^b^
Cause of death	Early Postoperative Complications	2.7% (*n* = 3)	3.6% (*n* = 2)	2.9% (*n* = 7)	2.0% (*n* = 1)	0.970^b^
	Cancer related	18.9% (*n* = 21)	20.0% (*n* = 11)	20.4% (*n* = 50)	17.6% (*n* = 9)	0.968^a^
	Non-cancer related	6.3% (*n* = 7)	5.5% (*n* = 3)	6.9% (*n* = 17)	1.9% (*n* = 1)	0.768^b^
	Uncertain	2.7% (*n* = 3)	3.6% (*n* = 2)	0%	1.9% (*n* = 1)	0.015^b^
Mean (95%CI) overall survival (months) excluding early postoperative complications		42 (42–45)	42 (38–47)	43 (40–46)	46 (41–50)	0.778^g^

*PPC* postoperative pulmonary complication, *ITU* intensive therapy unit, *LOS* length of stay.^a^ Chi-square test with Bonferroni post-hoc analysis;^b^ Fisher’s Exact test with Bonferroni post-hoc analysis. When overall *p*-value is less than 0.05; each subscript letter^(c-e)^ denotes a subset of smoking category whose column proportion does not differ significantly from each other (Bonferroni post-hoc multiple comparison test);^f^ kruskal-Wallis test with Dunn-Bonferroni post-hoc multiple comparison test;^g^ ANOVA with Bonferroni post-hoc analysis.;^h^ interquartile range not applicable due to number of patients

### Short-term outcome

Compared to never smokers, current smokers had a higher frequency of ITU admission (14% vs. 0%; *p* = 0.001) as well as longer median (IQR) hospital LOS (days) (6 [5] vs. 5 [2]; *p* = 0.001). Compared to current smokers, in the ex-smoking groups there was a trend for a lower frequency of ITU admission (<6 weeks, 5.5% vs. ≥6 weeks, 4.5%). but there was no significant differences between ex-smoking groups, or when compared to current and never smokers. Patients who stopped smoking ≥6 weeks prior to surgery were found to have a significantly shorter ITU median (IQR) LOS (days) compared to current smokers (3 [2] vs. 4 [10]; *p* = 0.002). There was no significant difference in 30-day hospital readmission rates between any of the groups. There were no significant differences in the 30-day mortality, 90-day mortality or causes of death (cancer or non-cancer related) between any of the groups.

### Long-term survival

**Fig. 1:**
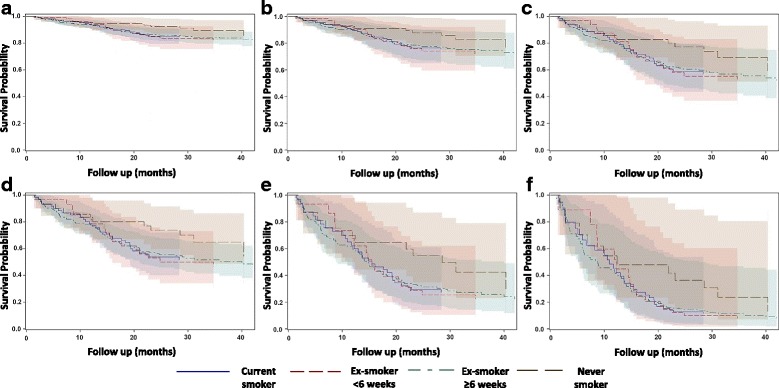
Predictive Survival curves with 95%CI for patients with different preoperative smoking status, excluding early postoperative deaths, adjusted for the significant covariates from the Cox proportional hazards model (Table 3). Covariates set at: **a**) PPC 0, Stage I; **b**) PPC 1, Stage I; **c**) PPC 1, Stage II; **d**) PPC 0, Stage IIA; **e**) PPC 0, Stage IIIB; **f**) PPC 1, Stage IIIB. Age and LOS were set at mean and median values respectively

When assessing for survival, the mean follow up time for all patients was 29.8 months (95%CI 28.4–31.1). After excluding early postoperative deaths, Cox regression analysis showed significant independent risk factors for late deaths following thoracic surgery to be PPC development, age, cancer staging and hospital LOS (Table 3). Smoking variable was not a significant predictor for late deaths (*p* > 0.05) but after adjusting for the significant covariates, the adjusted survival curves for the never smokers were clearly above the survival curves for the rest of smoking categories (Fig. 1).

**Table 3:** Independent risk factors for late deaths

Variables	Parameter estimate	SE	*p*-value	HR	95%CI
Stage (II vs. I)	1.11	0.22	<0.0001	3.03	1.97–4.71
Stage (IIIA vs. I)	1.35	0.25	<0.0001	3.87	2.39–6.28
Stage (IIIB vs. I)	1.88	0.61	0.0019	6.54	1.57–18.33
Age	0.03	0.01	0.0115	1.03	1.01–1.05
PPC	0.53	0.27	0.0469	1.71	0.98–2.82
LOS	0.03	0.01	0.0324	1.03	1.01–1.05

*PPC* postoperative pulmonary complication, *LOS* length of stay

## Discussion

This study demonstrates that patients who continue to smoke until the date of curative-intent lung cancer surgery have a higher postoperative morbidity, including higher frequency of PPC, longer hospital LOS, and a higher frequency of ITU admission. In ex-smokers there was trend for reduced frequency of PPC and ITU admission compared to current smokers, but there was no significant difference in observed outcome between patients who quit smoking less <6 weeks versus ≥6 weeks prior to surgery. We found no significant differences in early mortality or long-term survival between any of the groups within our follow up period.

Our study demonstrated that the incidence of PPC in current smokers is 22%, which is greater than 10 times that of never smokers and twice that of ex-smokers. Comparing this observed incidence with results from other studies is challenging because of the differences in the definition of PPC, which may extend to include potentially more severe yet less frequent complications such as pulmonary embolism, prolonged air leaks and bronchopleural fistulas [8]. Despite this, the frequency of PPC in smokers using the MGS is within the wide range of pulmonary events reported in other studies (6.9–43.2%) [5, 9]. Our finding of PPC to be higher in current versus never smokers is also supported by other studies [5, 10].

Despite the observed trend, our study showed no significant difference in PPC frequency between ex-smokers and current/never smokers. Other studies have found no significant difference in ex-smokers and current/never smokers which is most likely due to much smaller study sample sizes [9], and/or the exclusion of a never smoking control group [11, 12]. In a general thoracic surgery cohort, we recently demonstrated that compared to never smokers, ex-smokers were more than twice as likely to develop a PPC [2]. However, the timing of smoking cessation prior to surgery, and the subsequent effect of postoperative outcome was not investigated.

In our prospective cohort, using 6 weeks as a cut off, we were unable to find an optimum duration of preoperative smoking cessation to reduce PPC incidence. Despite this, our study has demonstrated that stopping smoking 6 weeks prior to surgery seems to reduce, and not increase PPCs compared to current smokers. Interestingly, the study by Nakagawa et al (*n* = 288) [4] found the incidence of PPCs among ex-smokers (2–4 weeks prior to surgery) was 53.8%, whilst in current smokers was 43.6%, which were both significantly higher when compared to the never-smokers (23.9%; *p* < 0.05). However, this study is limited by the retrospective nature of data collection. As defined by Russell Standard [13], the study found that at least 4 weeks of smoking abstinence was needed for a marked reduction in PPC (53.8% to 34.7%), whilst the incidence of PPC in patients who had a smoke-free period of 9–12 weeks or longer approached the same incidence as those in the never-smoked group. A large prospective review of the General Thoracic Surgery Database from the United States (*n* = 7990) found that the risk of PPCs following primary resection for lung cancer steadily declined with longer intervals of smoking cessation (4 days - 1 month, 1–12 months, >12 months) [5]. However the differences were not significant enough between the groups to recommend optimal interval of smoking cessation. One critique of this study is that the incidence of PPC was low (6.9% in smokers) as only major pulmonary events were reported, suggesting minor yet more frequent PPCs may have been missed.

Smoking had no direct effect on postoperative mortality or long-term survival during in our study. Nevertheless, the adjusted survival curves for never smokers were clearly above those of the other smoking categories especially after the first 12 months of follow up, suggesting a likely more favorable outcome even though this did not reach statistical significance. This may be in part due to the short follow up period or the smoking habits of patients after surgery; some may stop whilst others may restart. Other studies have shown increased mortality in smokers compared to never smokers, but no significant difference amongst the current and ex-smoking groups [5, 13]. The deleterious effect of continued smoking on long-term survival after being diagnosed with lung cancer is increasingly evident [14], but few papers have looked into the effect of preoperative smoking cessation on long-term survival in lung cancer patients undergoing curative-intent surgery. A multicenter review of 169 patients undergoing NSCLC resection found that current smokers had a reduced 5-year survival in comparison to never smokers (72% vs. 91%, *p* = 0.02), and that stopping smoking a year prior to cancer diagnosis had no significant difference in long-term survival [9]. Interestingly, although smoking was not an independent risk factor for late-deaths in our study, PPC development and LOS were independent risk factors for late-deaths, both of which were significantly increased in the current smokers.

We found that lower FEV_1_% predicted, BMI and COPD diagnosis were associated with current smoking on univariate analysis. Other investigators have found that the prevalence of low and normal BMI was greater among patients who are active smokers; those underweight patients had a greater risk-adjusted LOS compared to normal weight patients [15]. We have previously investigated the risk factors for developing a PPC, and although lower preoperative FEV_1_% predicted and COPD diagnosis are associated with PPC on univariate analysis, only COPD diagnosis is regarded as an independent risk factor for PPC development or multivariate analysis [1, 2]. Even in these models, current smoking remains the strongest independent risk factor for PPC development. Authors have found carbon monoxide diffusion capacity (DLCO) to be associated with PPC development [10]; however, DLCO was not included in this study, as it was only performed in patients with limited exercise tolerance so data are limited.

The observed effects of smoking and increasing frequency of PPC could be explained by the suppressive effect of cigarette smoking on the innate immune system. Smoking impairs the mucociliary escalator and the ability to remove foreign bodies/pathogens in the larger airways. This normalises after 15 days following smoking cessation implying that it is not the only etiological factor in PPC development [16]. Smoking seriously impairs antimicrobial [17, 18] and proinflammatory [19] functions of alveolar macrophages. Furthermore, there is a compounding effect on anaesthesia and smoking on suppression of intraoperative antimicrobial/phagocytic activity in alveolar macrophages. Smoking cessation of 6 months was needed to normalise proinflammatory function whilst only 3 months was required to normalise phagocytic function [20].

The question of whether a smoking cessation programme in surgery affects postoperative outcomes has been more recently reviewed [21]. Implementation 6–8 weeks prior to elective orthopedic surgery reduced postoperative complications (18% vs. 52%, *p* = 0.0003) [22], whilst implementation 2 weeks prior to colorectal surgery made no difference [23]. Despite increasing numbers of small RCTs suggesting intensive smoking cessation treatments work in people with pulmonary diseases such as lung cancer, many patients continue to smoke [24]. In thoracic surgery, pulmonary rehabilitation programmes have shown promise in increasing smoking cessation, with a trend for fewer PPCs in the intervention group [25]; however, RCTs are needed to confirm efficacy.

### Limitations

In this prospective study, the main limitation is the reliance on self-reporting smoking status in preoperative patients to ascertain smoking cessation, which could result in recall bias and underestimate the real smoking habits of patients. Use of biochemical confirmation by use of exhaled carbon monoxide testing and measuring cotinine levels from urine, saliva or blood could be used in future studies [26]. Furthermore, smoking cessation data was collected as a categorical variable, and therefore additional statistical analysis using ROC curve analysis could not be performed to further inform on a cut-off for the duration of smoking cessation needed to reduce PPC incidence. In our study, there were a low number of VATS cases in our prospective cohort (8.4%), and only 5.6% of patients with stage 1 NSCLC underwent VATS resection. The data of this study preceded the growth of VATS lobectomies in NSCLC patients; the vast majority of lung resection over this time frame was performed via open thoracotomy. This low level of VATS resections could confound the results and make them less generalisable considering that many studies have demonstrated a higher postoperative pulmonary complication (PPC) rate with open thoracotomy vs. VATS resections [27, 28]. Therefore, further studies are required to investigate the effects of smoking cessation on PPC frequency in VATS verses open thoracotomy cases.

## Conclusions

Our real-life study demonstrates that approximately 1 in 5 patients continue to smoke prior to curative-intent surgery for NSCLC. Smoking cessation prior to surgery reduces PPC incidence as well as other important markers of postoperative morbidity, thus we recommend that all patients should undergo formal smoking cessation as part of the routine work up. The optimum timing for preoperative smoking cessation however is yet to be defined. Further research into effective preoperative smoking cessation programmes and their short-term and long-term effects is urgently needed.

## Abbreviations

ASA: American Society of Anaethesiologists
BMI: Body mass index
CI: Confidence interval
COPD: Chronic obstructive pulmonary disease
CXR: Chest x-ray
DLCO: Carbon monoxide diffusion capacity
FEV_1_: Percentage predicted forced expiratory volume in one second
HDU: High-dependency unit
IQR: Interquartile range
ITU: Intensive therapy unit
LOS: Length of stay
MGS: Melbourne group scale
NSCLC: Non-small cell lung cancer
PPC: Postoperative pulmonary complication
VATS: Video-assisted thoracoscopic surgery
